# An Assistive Soft Wrist Exosuit for Flexion Movements With an Ergonomic Reinforced Glove

**DOI:** 10.3389/frobt.2020.595862

**Published:** 2021-01-18

**Authors:** Domenico Chiaradia, Luca Tiseni, Michele Xiloyannis, Massimiliano Solazzi, Lorenzo Masia, Antonio Frisoli

**Affiliations:** ^1^Percro Laboratory, Tecip Institute, Sant'Anna School of Advanced Studies, Pisa, Italy; ^2^Sensory-Motor Systems (SMS) Lab, Institute of Robotics and Intelligent Systems (IRIS), ETH Zurich, Switzerland and the Spinal Cord Injury Center, University Hospital Balgrist, Zurich, Switzerland; ^3^Institut für Technische Informatik (ZITI), Heidelberg University, Heidelberg, Germany

**Keywords:** soft wrist exosuit, ergonomics, assistive robot, flexible exoskeleton, admittance control, cable-driven exosuit, wearable robotics, electromyography

## Abstract

Soft exosuits are a promising solution for the assistance and augmentation of human motor abilities in the industrial field, where the use of more symbiotic wearable robots can avoid excessive worker fatigue and improve the quality of the work. One of the challenges in the design of soft exosuits is the choice of the right amount of softness to balance load transfer, ergonomics, and weight. This article presents a cable-driven based soft wrist exosuit for flexion assistance with the use of an ergonomic reinforced glove. The flexible and highly compliant three-dimensional (3D)-printed plastic structure that is sewn on the glove allows an optimal force transfer from the remotely located motor to the wrist articulation and to preserve a high level of comfort for the user during assistance. The device is shown to reduce fatigue and the muscular effort required for holding and lifting loads in healthy subjects for weights up to 3 kg.

## 1. Introduction

Exoskeletons are physical human-robot interfaces (HRIs) conceived for several purposes such as rehabilitation, assistance, and haptic interaction in virtual or remote environments (Pons, 2008). The robotic community put a strong effort into developing wearable powered robots since at least 1967 (Mosher, 1967) when one of the earliest exoskeleton for motion augmentation was presented. Gradually, the same principles were translated into the rehabilitation and assistance field to aid functional substitution in patients suffering from motor disorders. Only in the past years, commercial solutions entered the market (Guizzo and Goldstein, 2005) to assist workers and reduce fatigue and musculoskeletal strain in burdensome and repetitive tasks (Kim et al., 2018). For instance, in automotive manufacturing, upper body exoskeletons have become a new fast-growing frontier for enhancing productivity, ergonomics, and safety of workers. Commercially available solutions include, among others, the Paexo by Ottobock, EksoVest by Ekso Bionics, MATE by Comau, and ShoulderX by SuitX. Most of the proposed solutions make use of a rigid kinematic structure parallel to the human one to allow the wearer's motion, mainly because of the payload for which they are conceived. However, rigid exoskeletons can introduce joint misalignment that causes discomfort, restrict movements, or, in the worst case, cause pain (Jarrassé and Morel, 2012).

A strategy to overcome joint misalignment is to interpose passive Degrees of Freedom (DoF) between the human limb and the exoskeleton anchor point (Stienen et al., 2009). However, this solution increases the weight, the complexity, and the cost of the robot. An alternative approach is using soft materials, such as fabric, rubber, and thermoplastics, to transmit forces and torques to the human body. Due to their high compliance that allows considerable displacements, soft wearable robots, also called exosuits, virtually have an infinite number of DoF and, therefore, do not suffer from joint misalignment and their interference with the complex natural biomechanics of human movements is intrinsically limited by design. Soft exosuits thus represent a valid and promising solution to assist human limb in work environments, as well as for domestic assistance, or recovery of limb functions. What is more, when operations involve small loads (up to 3 kg) and repetitive actions, the advantages of light and soft exosuits over their rigid counterparts become even more relevant. For example, cable-driven exosuits allow locating the actuation stage where its additional weight has the least metabolic impact on its wearer (Chiaradia et al., 2018b) and they can provide similar level of assistance of rigid exoskeletons.

Nowadays, most of the commercial exoskeletons for assistance to workers focuses on the proximal articulations of the upper limb, i.e., shoulder flexion/extension, and abduction/adduction. Some of the factors that discourage the implementation and adoption of full-limb solutions are the encumbrance, the weight, and the cost of these complex solutions; consequently, the provided assistance is only partial. This work aims at proposing a light, soft, and ergonomic solution for the wrist assistance that can be combined with shoulder/elbow exosuits. In fact, the proposed wrist exosuit is designed as a module of a complete upper limb exosuit where existing solutions for the shoulder (Tiseni et al., 2019), the elbow articulation (Chiaradia et al., 2018a; Xiloyannis et al., 2019a), and the hand (Xiloyannis et al., 2019b) will be joined.

**Figure 1:**
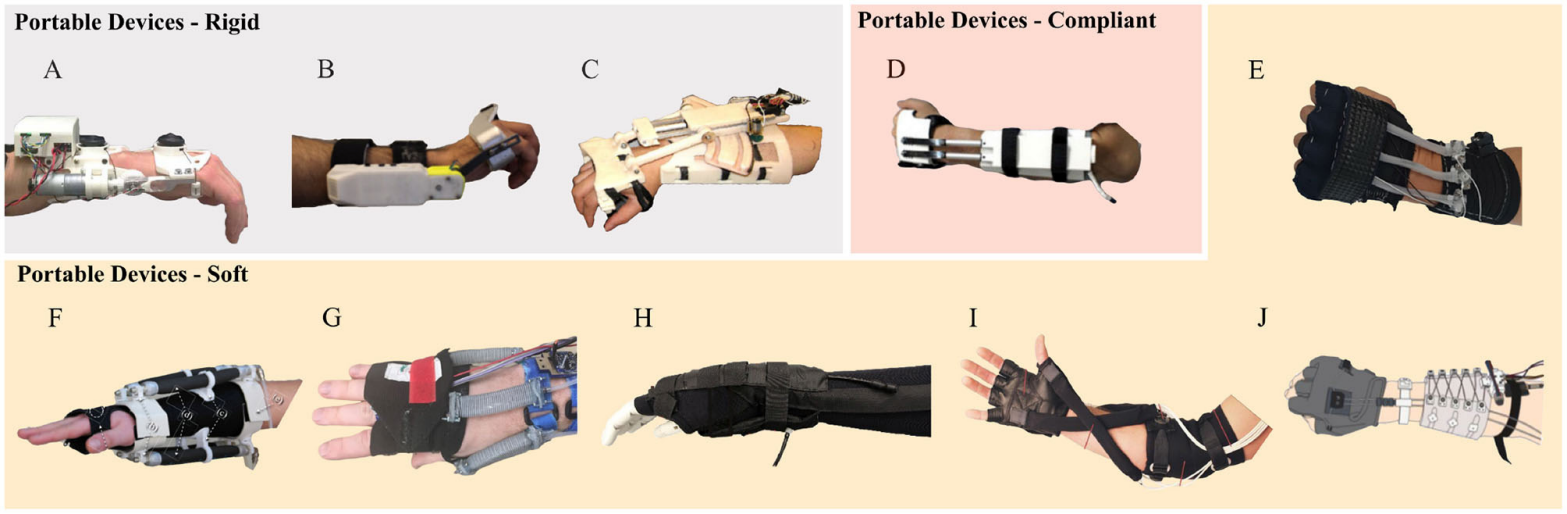
State of the art of portable wrist exoskeletons can be grouped in rigid **(A–C)**, compliant **(D)**, or made of soft materials **(E–J)**. Main intended use of portable exoskeletons are rehabilitation and assistance.

In literature, several wrist exoskeletons were proposed and they can be grouped in “stationary” or “portable” solutions. Stationary wrist exoskeletons are mostly conceived for rehabilitation purposes or general haptics, and each of them exploits a specific feature concerning the kinematics, the mechanics, or the power transmission. Stationary exoskeletons are design to be rigid while preserving low inertia and friction and can be implemented by gearmotors (Cappello et al., 2015), cable transmission (Perry et al., 2007; Pehlivan et al., 2014; Pezent et al., 2017), differential cable transmission to enhance compactness (Buongiorno et al., 2018), and parallel structure to enhance rigidity (Gupta et al., 2008; Martinez et al., 2013). Figure 1 shows a state of the art on portable wrist exoskeletons. Differently from stationary exoskeletons, portable solutions offer the possibility to assist the user more naturally, also in complex tasks that require walking or are performed in large workspaces. State of the art rigid and portable solutions focused on supporting wrist extension only (Lambelet et al., 2017) (in Figure 1A), flexion/extension (Sangha et al., 2016) (in Figure 1B) or both flexion/extension and ulnar/radial deviation (Khokhar et al., 2010) (see Figure 1C) with applications in the rehabilitative field. Despite these solutions being portable and weighing less than their stationary counterparts, they do not provide a way to mitigate joint misalignment. An interesting implementation is proposed in Higuma et al. (2017) (in Figure 1D). They developed a portable compliant wrist exoskeleton equipped with two elastic elements that largely deform when forces are transmitted from the forearm linear actuation module to the wrist. The device assists flexion/extension and adduction/abduction of the wrist with the advantage of being compact, light, and inherently flexible.

Still, soft exosuits can offer the possibility of reducing weight and enhancing ergonomics due to their mechanical features. The SWA wrist exosuit (Jeong et al., 2019) (in Figure 1E) is a shape memory alloy (SMA)-based wearable robot that assists 2 DoF of the wrist motion and it is based on coil spring-shaped SMA that acts like a muscle. It is a light solution (weight 151 g) and it can exert a maximum force of 10 N. Despite this solution is completely flexible, the SMA actuators offers a limited bandwidth for industrial exosuits due to the need of heat dissipation that underlie its physical working principle. The 2 DoF EXOWRIST (Andrikopoulos et al., 2015) (Figure 1F), which is driven by pneumatic muscle actuators, is a safe and reliable exosuit that exploits ergonomics by using a neoprene-based glove to provide the necessary orthopedical support and protection to the wrist.

A pneumatic wrist exosuit conceived for haptic feedback (Skorina et al., 2018) (Figure 1G) uses pressure-driven soft actuators called reverse pneumatic artificial muscles that are originally pre-strained and release compressive stress under pressure. It is driven by a precise pulse width modulation control of the soft actuators, which makes the device suitable for real-time haptic feedback applications. A medical application of a pneumatic robotic sleeve that dynamically adjust the position of the wrist in real time to a neutral angle to prevent or release CTS strains is presented in Zhu et al. (2017) (Figure 1H) This device is created using a sturdy, elastic fabric sleeve, and two heat sealable thermoplastic airbag actuators that assist the flexion/extension of the wrist. A McKibben-type actuation, in conjunction with a fabric glove, an elbow sleeve, and a tensioning mechanism, was chosen instead in Bartlett et al. (2015) (Figure 1I). Despite pneumatic actuation offers a safe and extremely soft solution, its need for pressurized air makes it difficult for them to be adopted in untethered devices for industrial usage.

A device designed with particular attention to ergonomics is the Exo-Wrist (Choi et al., 2019) (Figure 1J), a soft cable-driven exosuit that assists the wrist of a paretic arm in performing the dart-throwing motion (DTM) with an active forearm anchor. This exosuit prevents medical issues due to long-term pressure by using an active anchor that compresses the body only when anchoring is needed. The Exo-Wrist design focus mainly on the ergonomics of the forearm module and it finds in rehabilitation and assistance in medical fields its target application. Thus, we found a slight room for improvement on the hand module design conceived for the industrial field by focusing on the necessity of a flexible interface, where its stiffness is a trade-off between force transmission efficiency and ergonomics.

The analysis of the literature combined with the typical needs of industrial operators performing repetitive tasks suggests the investigation of a remotely actuated solution to preserve lightness and keep dynamical loads low that can satisfy ergonomic and practical requirements. Also, optimization of force/torque transmission is a crucial aspect of the exosuit adoption in real work scenarios.

In the literature, there are only few devices that implement hybrid solution—rigid and soft—to optimize force transmission, such as the palm bars in devices for the hand like the Roboglove (Diftler et al., 2015) and SPAR Glove (Rose and O'Malley, 2018), which have used softgoods embedded with rigid three-dimensional (3D)-printed components. Moreover, the Roboglove uses rigid saddles around the fingers to distribute the force from the tendon evenly and to prevent cinching of the cable loop around the finger, whereas the SPAR Glove uses rotating mechanisms such as the BOA's on Armstrong to fasten cuffs/gauntlets. The ExoGlove PM (Yun et al., 2017) balance soft and rigid elements in the finger modules: it uses 3D-printed spacers to hold the distance between two actuator modules at certain required values that robust enough to transmit large forces. Finally, in Kadivar et al. (2017) a hybrid solution for the elbow and shoulder joints is presented in a preliminary study.

In this work, we present a first prototype of a wrist soft exosuit intended for healthy subjects assistance in work environments. It is provided with a glove that was reinforced with flexible 3D-printed plastic components. These flexible parts were designed to be as stiff as needed to efficiently transmit forces and preserving comfort due to their ergonomic shape. Due to the sewn reinforcement, the obtained exosuit was light, safe, and effective in assisting users in both isometric and dynamic tasks using an admittance controller. The use of the flexible structures combined with the fabric glove was of relevant importance for the correct delivery of exosuit assistance as highlighted by the significant reduction of users muscular activity and fatigue obtained in the conducted experiments.

## 2. Materials and Methods

### 2.1. Exosuit Design

#### 2.1.1. Overview

**Figure 2:**
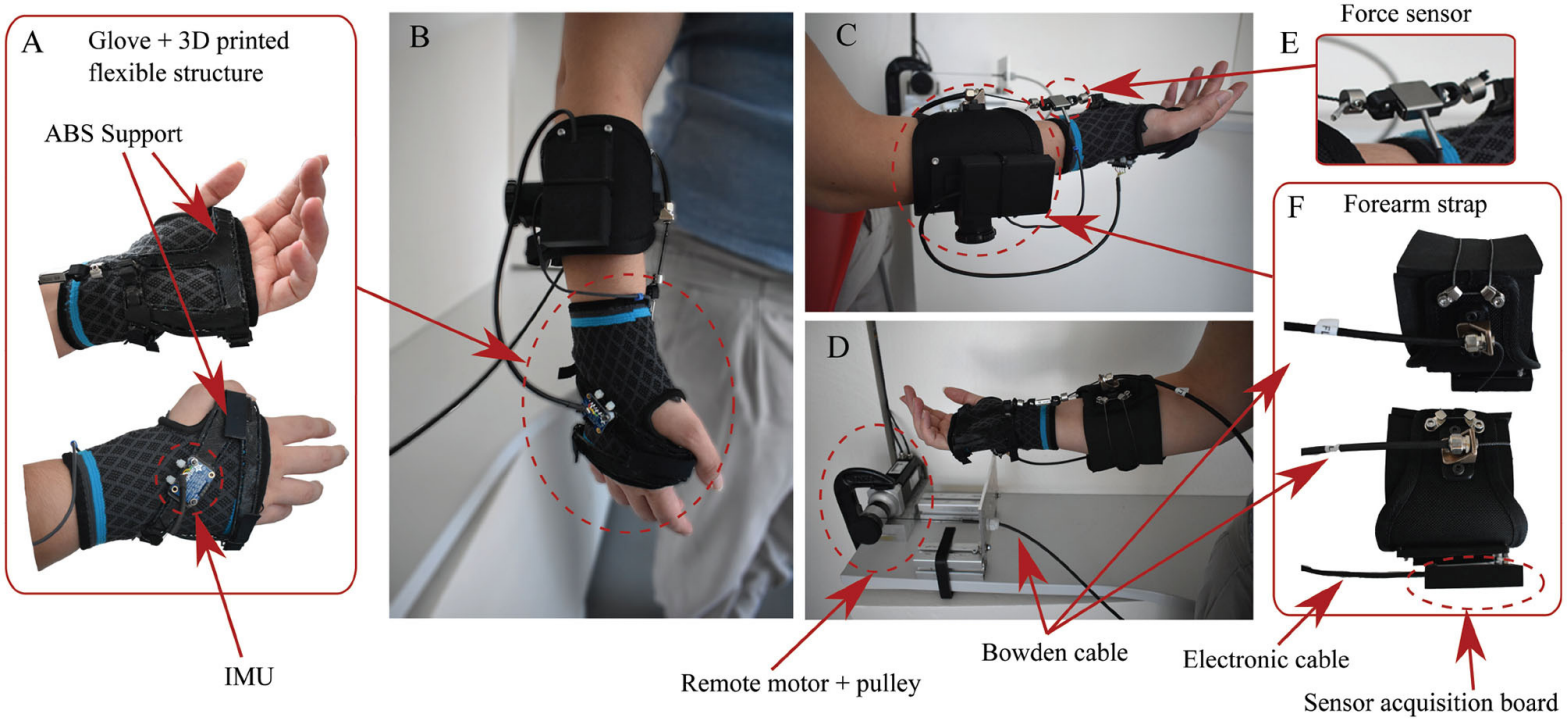
Design of the soft exosuit of the wrist **(B–D)**. The exosuit comprises a glove that is reinforced through a flexible 3D-printed structure **(A)** and a strap that wraps around the forearm **(F)**. These two act as anchor points: the Bowden cables' outer sheath is attached to the forearm strap and the inner tendons to the glove palm's end. A load cell **(E)**, an encoder, and an inertial measurement unit **(A)** sense the interaction force, the wrist flexion angle, and angular speed. The actuation stage comprises a brushless motor, equipped with a gearhead and encoder, that drives a spool around which the suit's tendons are wrapped **(D)**. For this first prototype, the actuation stage was fixed on a table.

The soft wrist exosuit was designed to assist the flexion of the wrist in applications that involve repetitively small weight moving up to about 3 kg. We targeted the flexion movement by analyzing typical postures of workers' upper limb while holding or moving objects with two hands, for example, box loading and unloading in logistic work environments. The exosuit consists of a mix of soft and rigid materials combined to achieve ergonomic and functional design. As shown in Figure 2, the exosuit is composed of two wearable parts: the forearm strap, a proximal one, located on the forearm, and the glove, a distal one, located on the hand. It is actuated by a remotely located motor through a Bowden cable transmission unit and it is provided with an inertial measurement unit and a force sensor. The total weight of the wearable parts is approximately 0.3 kg (80 g for the glove and 215 g for the forearm strap), and the system can provide up to 3.0 Nm of estimated assistance torque to wrist flexion in any configuration. This rated assistance torque is sufficient to lift weight of about 3 kg, considering the weight located at the center of the palm, about 0.1 m from the wrist joint axis. As the proposed wrist exosuit does not rely on an external rigid kinematic chain parallel to the human one, it does not impose any constraints on the human motion, also causing possible discomfort in case of joint misalignment. This feature enhances the device's ergonomics and allows the wrist to move within a range of motion (ROM) of 150° (70° for dorsiflexion and 80° for extension) that is above the required ROM needed to perform activities of daily living (ADL) as reported in Ryu et al. (1991).

#### 2.1.2. Glove

The wearable glove module, shown in Figure 2A, represents the main novelty of this wrist exosuit design. It is made up of three parts: a soft orthosis for the wrist, made of elastic fabric, and two 3D-printed ABS supports, one for the palm and one for the back of the hand, that are stitched to the soft orthosis and can be tightened together through velcro straps. The anchor point for the tendon is located on the proximal part of the palm support. The two ABS supports allow the glove to bear cable tension without sliding over the hand and avoiding excessive displacement for the soft orthosis. Therefore, the cable tension is more efficiently converted into assistance torque for the wrist.

**Figure 3:**
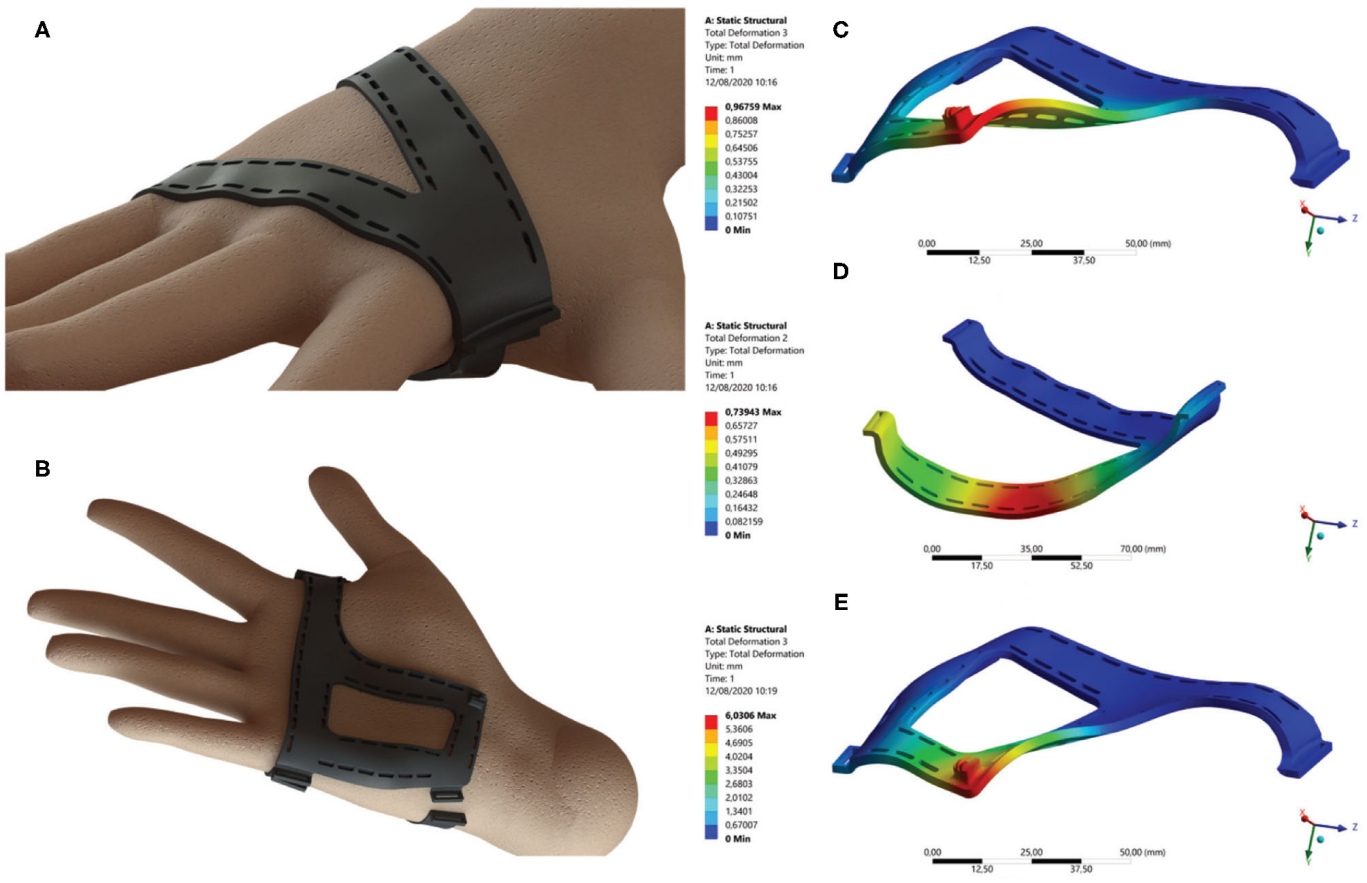
**(A,B)** CAD renderings of the three-dimensional (3D)-printed ergonomic supports. **(C,D)** Fem analysis with a load of 10 N applied on the supports by the hand. Estimated average stiffnesses: **(C)**: 10.33 N/mm; **(D)**: 13.53 N/mm. **(E)**: Fem analysis with a load of 80 N applied on the support by the cable tendon. Estimated average stiffness: 13.27 N/mm. Stiffnesses values are compatible with the needed mechanical performance but, at the same time, allow the supports to be compliant enough to compensate for misalignment errors.

Ergonomics is the main design goal for the soft wrist exosuit. The ABS supports are designed to this end to allow a safe and comfortable force transmission from the tendon to the wrist joint. In fact, the design of the supports started from a 3D scan of the right hand, as shown in Figures 3A,B. The hand's surfaces have been used to build the ABS parts, which were then conveniently shaped to avoid excessive stiffness while keeping their functionality. In particular, the design of the two supports is driven by two main functional requirements; the first one is not limiting the ROM of the wrist joint and the fingers, whereas the second one is ensuring a comfortable load transfer between the exosuit and the user. For this reason, the design tries to redistribute a force and a moment at least on two large surfaces both on the dorsal support and on the palm support, but it is necessary to avoid limiting the thumb motion. Moreover, two full shells would cover the whole hand, badly affecting the capability of feeling touch for the user. These considerations led to the design of the V-shape for the dorsal part, while, for the palm support, we modified the V-shape to support the tendon anchor point properly.

The main idea behind this choice is achieving a conforming contact between the ABS supports and the hand to avoid localized pressure peaks, which notoriously cause discomfort for the user (Jarrassé and Morel, 2012). The thickness of the plastic reinforcement and its geometrical characteristics were chosen first to confer flexibility under flexural loads and second to ensure enough robustness to bear the cable tension. Both features were verified in the design phase via finite element (FEM) analysis (see Figures 2C–E). Flexibility was verified by simulating a load of 10 N applied on the supports by the hand. The estimated average stiffness of the palmar support was 10.33 N/mm, whereas the dorsal support stiffness was D 13.53 N/mm. Robustness of the palmar support was evaluated by simulating a load of 80 N applied on the support by the cable tendon. The estimated average stiffness was equal to 13.27 N/mm. Stiffness values are compatible with the needed mechanical performance and allow the supports to be compliant enough to compensate for misalignment errors.

#### 2.1.3. Forearm Strap

The forearm strap, as shown in Figure 2F, consists of an adjustable U-shaped bracelet made of fabric and reinforced with a thin metallic foil. A cable that can be stretched using a rotating mechanism tightens the U-shaped bracelet to the forearm in order to avoid relative motion between the strap and the forearm. The forearm module is not designed to rely on shear forces only to keep its position on the forearm, indeed the wrist exosuit module is intended to be attached through a cable to the elbow exosuit module and in general to the fully upper-limb exosuit. To distribute pressure on the forearm due to reaction forces, we interposed a 5 mm thick neoprene padding between the strap and the user's forearm.

#### 2.1.4. Actuation Unit

The actuation unit, as shown in Figure 2D, consists of a Kollmorgen Motor (AKM23F) with an Apex Dynamics planetary gear drive —PG II 040, transmission ratio 1:10, on whose exit shaft an ABS pulley—45 mm diameter—is connected. The pulley drives a Dyneema cable—multi-stranded kevlar, 1.5 mm diameter, 136 kg max. load—that passes through a Bowden sheath (Shimano SLR, diameter 5 mm). The Bowden sheath is fixed to ground on one side and to the forearm strap on the other side, and pulls the glove, providing assistance torque to the wrist flexion. For this first prototype, the motor is connected to the test bench; nevertheless, a more compact torque motor will find its final location in the backpack allowing wrist module integration in a mobile upper limb exosuit.

#### 2.1.5. Sensors

The soft wrist exosuit is equipped with a 9 DoF inertial measurement unit (IMU) (BNO055, Bosch) fixed on the ABS support on the hand's back, and with a load cell (Futek LSB25), which provides cable tension measurement (see Figure 2E). In particular, the IMU is used to obtain an estimate of the wrist flexion angle by the on-chip sensor fusion algorithm and the estimate of the flexion/extension angular speed from the gyroscope reads. It is worth mentioning that the estimation of the wrist flexion angle in a generic upper limb movement required a second IMU fixed on the forearm and belonging to the elbow exosuit module. The angle estimate is then obtained using rotational matrix mathematics.

### 2.2. Control Strategy

**Figure 4:**
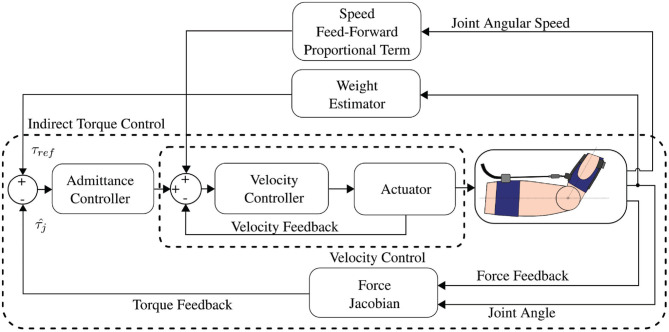
Schema of the admittance controller for transparency and gravity compensation. An outer admittance control loop tracks a reference profile that is the sum of two contributes: the first compensates for the gravity of the arm and of the load, and the second improves transparency by providing the torque needed for human movement following. The admittance controller computes a motion reference as an interaction torque is sensed, according to the admittance specified by a PID controller. The inner velocity loop is tuned to be as stiff as possible to reject force disturbances like stiction and backlash.

**Figure 5:**
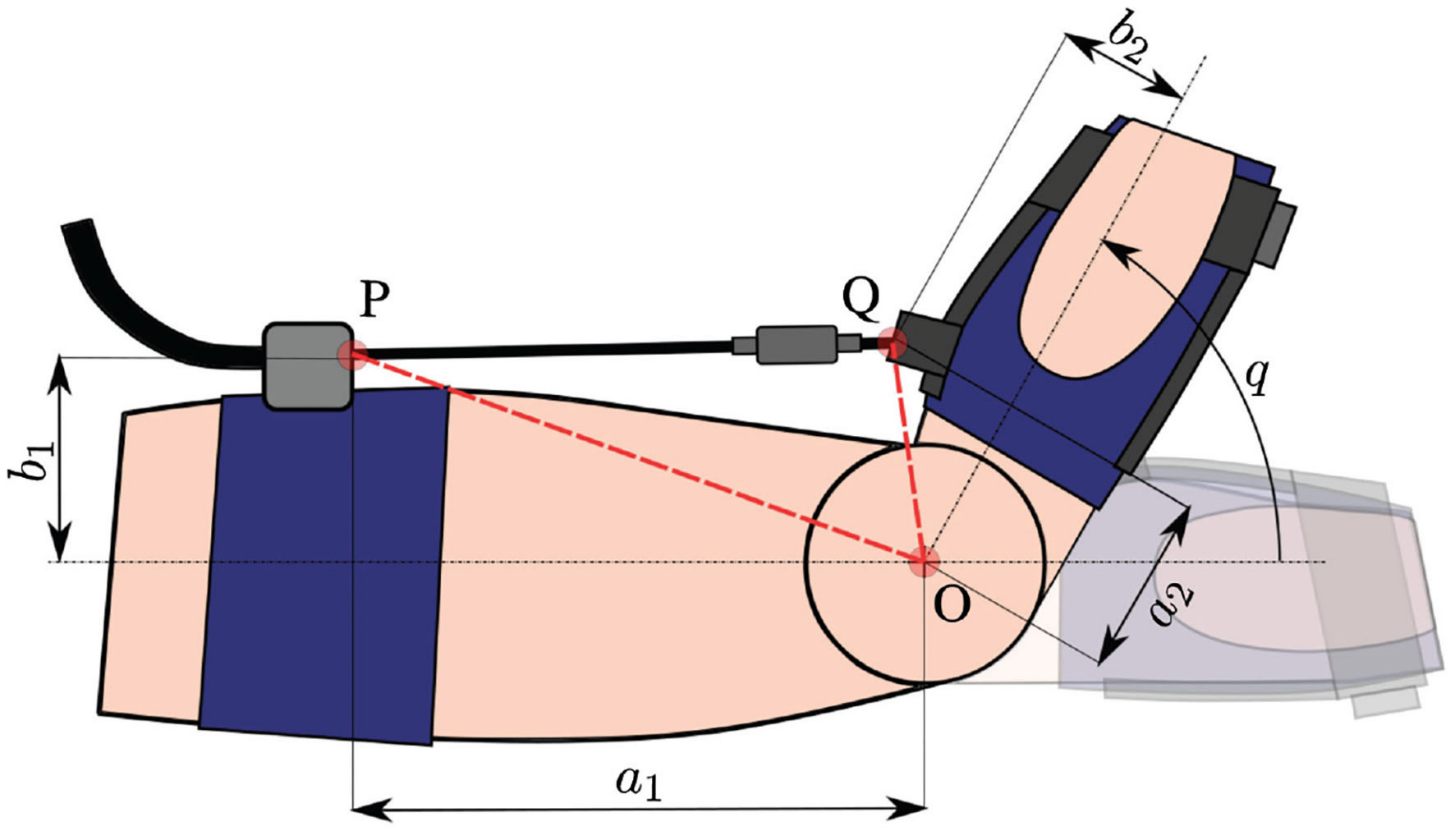
System model: *q* is the joint angle; O represents the joint rotation axis, while P and Q are the cable anchor points; *a*_1_, *a*_2_, *b*_1_, and *b*_2_ are estimated geometric parameters useful to define anchor points coordinates.

The device is controlled using an admittance controller implemented in an embedded PC, CongaTech PA5, running MATLAB Simulink Real Time at 5 kHz frequency, while a custom-made driver is used to command the motor torque. The admittance controller is shown in Figure 4. The inner control loop consists of a proportional-integrative-derivative (PID) controller setting the motor speed, with the velocity feedback coming from the motor encoder. The reference velocity for this loop is given by the outer control loop, consisting of an admittance controller. The reference torque is given by a weight estimator that considers both total weight value—hand's weight plus and additional weight—and the joint angle. The force feedback from the load cell is converted into estimated torque feedback through the force Jacobian, a scalar whose value depends on the joint angle. Figure 5 shows the 2D model that allows calculating the force Jacobian. Defining the estimated joint torque as $\hat{\tau_{j}}$, the cable tension as *f*, and the force jacobian as *J*_*F*_, it is possible to write:

(1) $$\begin{array}{r} {\hat{\tau }}_{j}=J_{F}f=|\text{OQ}\wedge \frac{\text{QP}}{\left|\text{QP}\right|}|f \end{array}$$

Given that:

(2) $$\begin{array}{r} \text{OP}={\left(a_{1},b_{1}\right)}^{T}\\ \text{OQ}={\left(a_{2}c_{q}-b_{2}s_{q},a_{2}s_{q}+b_{2}c_{q}\right)}^{T}\\ \text{QP}=-\text{OQ}+\text{OP} \end{array}$$

where *c*_*q*_ and *s*_*q*_ mean *cos*(*q*) and *sin*(*q*), respectively, and the force Jacobian can be calculated as:

(3) $$J_{F}=\frac{-a_{1}a_{2}s_{q}-b_{1}b_{2}s_{q}-a_{1}b_{2}c_{q}+a_{2}b_{1}c_{q}}{\sqrt{{\left(a_{1}-a_{2}c_{q}+b_{2}s_{q}\right)}^{2}+{\left(-b_{1}+a_{2}s_{q}+b_{2}c_{q}\right)}^{2})}}$$

The parameters values used for this application are shown in Table 1. Combining equations 1 and 3, the force signal from the load cell is converted into torque feedback for the admittance controller. Regarding the reference torque, the controller aims at compensating the torque exerted on the wrist joint by a nominal weight. Defining *m* as the total mass—hand mass plus eventually an additional weight—the reference torque is:

(4) $$\begin{array}{r} \tau_{ref}=mgc_{q} \end{array}$$

**Table 1:** Geometrical parameters used for the calculation of the force Jacobian.

**Parameter**	***a*_1_**	***a*_2_**	***b*_1_**	***b*_2_**
Value [m]	0.22	0.08	0.05	0.05

The admittance controller implements an additional feed-forward contribution to enhance the exosuit transparency. This contribution is directly proportional to wrist flexion angular speed, thus allows capturing the motion intention of the user and assisting the user's motion faster than using the gravity compensation alone. The optimal gain value for this contribution is characteristic of the system and does not change over participants.

### 2.3. Experimental Setup and Protocol

**Figure 6:**
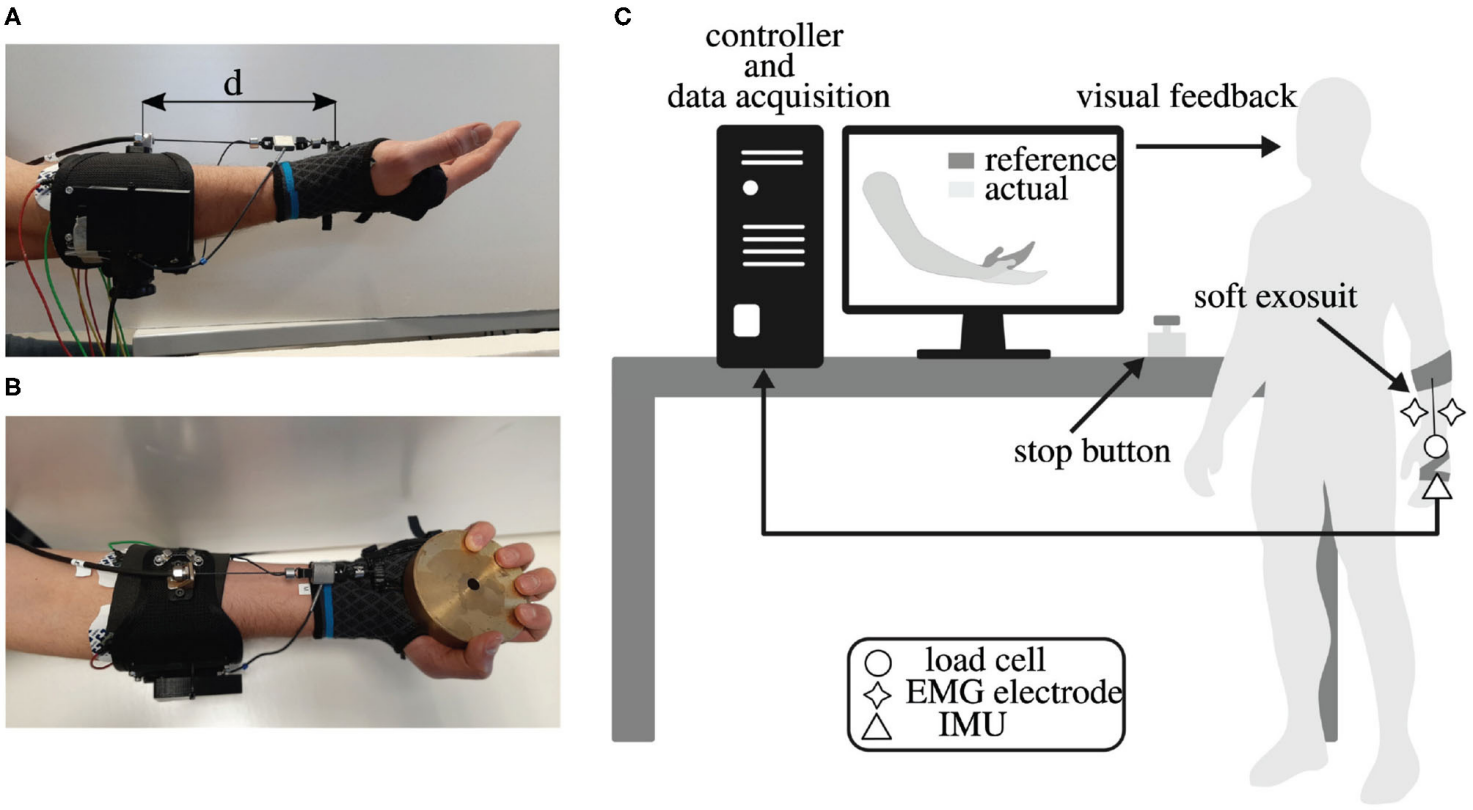
Experimental setup. **(A)** In the transmission stiffness test, two infrared markers were positioned on the anchor point of the forearm strap and of the glove. The distance *d* between the two markers, identified by the arrows, has been used to evaluated the anchor point stretching [it is equal to *d*(*t*) − *d*_0_, where *d*_0_ is the distance when no force was applied on the glove]. **(B)** During the isometric and the dynamic test, the user was asked to hold a 1.5 kg weight. **(C)** Subjects were asked to follow a reference trajectory displayed on a screen in the form of a moving hand, and the position of their own arm was superimposed to provide visual feedback. This was done in both the powered and unpowered conditions while monitoring the wrist flexion angle, the interaction force (only powered), and electromyography (EMG) activity of the four antagonistic muscles driving the joint.

Three experiments were conducted in order to assess the performance of the soft wrist exosuit under both isometric and dynamic conditions; in addition, a stiffness test was also performed to evaluate the transmission performance. The stiffness test aims at evaluating the capability of the glove of bearing the cable tension without excessively deforming, with and without the 3D-printed plastic reinforcement. The transmission displacement was measured using an optical motion tracking system (Optitrack V120: Trio) with passive infrared reflective markers. The isometric and the dynamic tests were performed to evaluate the physiological and kinematic effects of the assistive device on its wearer. The scheme of the experimental setup is depicted in Figure 6. A multi-channel electromyography (EMG) system (g.USBamp RESEARCH from g.Tec) has been used to record the activation signals of 4 muscles contributing to wrist flexion and extension: flexor carpi radialis (FCR) and flexor carpi ulnaris (FCU) for the flexion movement, and the extensor carpi ulnaris (ECU) and the extensor digitorum (ED) for the extension movement, as in Khokhar et al. (2010). Electrodes placement followed the SENIAM guidelines.

In the isometric and dynamic test, the participants perform the tasks with and without assistance (we refer to powered and unpowered condition). It is worth mentioning that, in the condition without the exosuit assistance, the cable tendons were unhooked from the distal anchor point to avoid hindering of the movement, and the motor's power source was turned off. We conducted a pilot study on four healthy participants (all males, aged 28.0 ± 4.0). All the participants signed a written consent in accordance with the Declaration of Helsinki on research involving human subjects. All participants presented no evidence or known history of skeletal or neurological diseases and exhibited normal wrist ROM and muscle strength.

#### 2.3.1. Transmission Stiffness Test

This test aims at evaluating transmission performance and thus validating the ergonomic design of the soft wrist exosuit. To this end, a comparative glove was built, starting from the same soft orthosis but without the plastic reinforcements. Therefore, the comparative glove has the anchor point for the cable directly attached to the soft material. We designed a task, repeated both with the reinforced glove and the comparative glove, where the participant, sitting and wearing the soft wrist exosuit, was asked to keep their forearm and their hand aligned parallel to the ground while the cable tension was progressively increased. The participant could grasp a handle to help themselves to hold still and oppose resistance to the cable tension. During the task, the Optitrack system has been used to measure and record the transmission stretching in the two conditions (reinforced glove and comparative glove), thanks to the two markers fixed to the two anchor points of the cable (forearm strap and glove, as in Figure 6).

#### 2.3.2. Isometric Test

It is known that myoelectric manifestations of muscle fatigue appear in the time domain as an increase in the EMG amplitude (Merletti and Parker, 2004). For this reason, we conducted a test to quantify the effect of the soft wrist exosuit on the onset of fatigue. In the isometric test, the participants were asked to keep their forearm and hand aligned horizontally, parallel to the ground, for 3 min while holding a brass weight—1.5 kg—with their hand. The load was chosen to be within the exosuit admissible assistance range (0–3 kg) and to allow the user to hold the weight without excessive discomfort, for 3 min, also in case of no assistance. Although fatiguing protocols often involve higher loads and isometric contractions until voluntary exhaustion (Potvin and Bent, 1997), our suit was not designed to bear high weights. This was done both for the powered and unpowered condition in a randomized order to avoid order effect. The task was repeated both with and without the exoskeleton. During the task execution, EMG signals were recorded.

#### 2.3.3. Dynamic Test

In the dynamic test, the participant was asked to track a Minimum Jerk Trajectory (MJT) that involves a wrist flexion movement from 0° to 60° at two different speeds—35 and 70°/*s*—with “w” and without “w/o” exosuit assistance, while holding a brass weight (1.5 kg) with their hand, performing a total of 15 repetitions. The MJT was used for its similarity to the movements of healthy subjects (Flash and Hogan, 1985).

During test execution, users visualized their own wrist flexion angle as a superimposed translucent replica of the reference one on a screen. To ensure participants were moving at the desired velocity, they were asked to match the movement of the character on the screen as accurately as possible. During task execution, both the EMG signals and the estimated flexion angle were recorded. As in the isometric experiment, the two conditions trials—powered and unpowered—were performed in a semirandomized order.

### 2.4. Data Analysis and Statistical Analysis

EMG signals were processed to extract its linear average envelope through a high-pass filtering (35 Hz) with a second-order Butterworth filter, full-wave rectification, and low-pass filtering (4 Hz, second-order Butterworth filter), and normalized using the individual MVC (Clancy et al., 2002). The root mean square (RMS) of the processed EMG signal was used as an index of the level of muscle activation. The effect of the exosuit on the onset of fatigue was evaluated through the rate of change of the RMS of the EMG values during the 3 min of isometric contraction. In particular, we used the average rectified value (ARV) of the EMG amplitude as indexes of fatigue, evaluated on epochs of 5 s. Then, we calculated their slope by fitting a first-order model with a least square method: a steeper positive slope for the ARV indicates a faster onset of fatigue.

Motor encoder angle, IMU flexion angle and speed, and load cell force signals underwent low-pass filtering at 10 Hz. For the quantification of the movement accuracy, we evaluated the coefficient of determination (r^2^) and the RMS error between the measured and reference trajectory.

We checked that the metrics were normally distributed using a Shapiro–Wilk test with a significance level of α = 0.05. All metrics resulted not normally distributed except for the ARV index. Non-normally distributed metrics were evaluated by a non-parametric Wilcoxon signed-rank test between the powered and unpowered conditions; our null hypothesis being that both samples came from distributions with equal mean. The normally distributed metric—the ARV—was statistically compared with a paired *t*-test (α = 0.05) between the powered and unpowered conditions.

Reported values and measurements from here onwards, in both graphs and text, are presented as mean ± standard error of the mean (SEM).

## 3. Results

### 3.1. Transmission Stiffness Test

**Figure 7:**
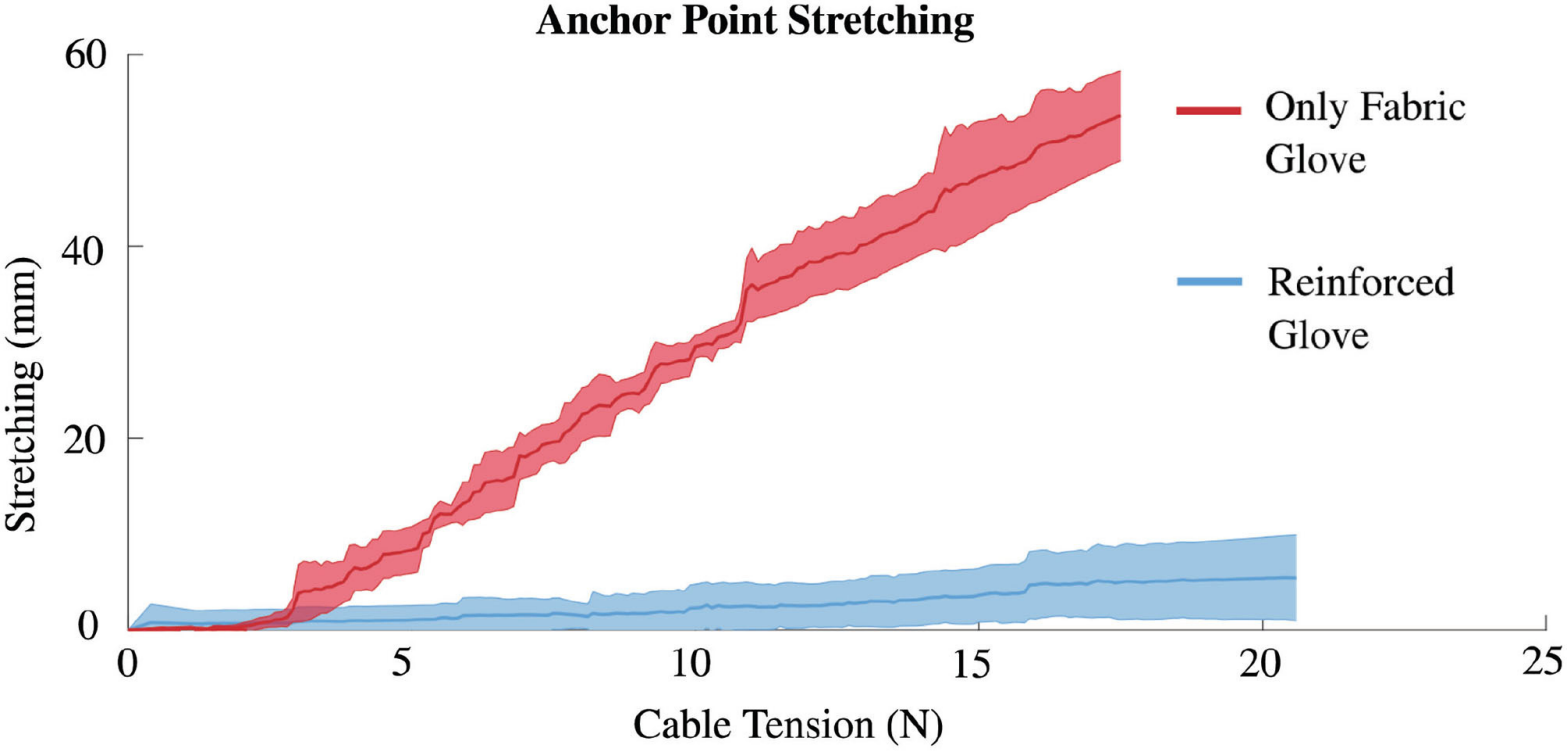
Ergonomics evaluation: The plot reports the stretching of the glove—averaged over subjects and trials—when no reinforcement was sewn (in red) and when the three-dimensional (3D)-printed plastic modules were sewn (in light blue). Without reinforcement, the transmission exhibited a stiffness of 0.296 N/mm and the anchor point shifted of more than 50 mm under a load of 17.5 N, thus causing discomfort due to fabric over stretch. In this case, it was not possible to continue with higher loads due to reaching of maximum contraction of the transmission cable. Reinforced glove solution exhibited a stiffness of 3.33 N/mm that was sufficient to comfortably transfer loads up to 50 N.

Figure 7 shows the result of the transmission test. The plot reports the stretching of the glove averaged over both participants and trials in the two configurations of the glove: without sewn reinforcement (in red) and with sewn reinforcement (in light blue). The noticeable stiffness difference in the two configurations is related to the efficacy of glove force transmission. The estimated stiffness of the glove without reinforcement—computed by linear regression of data—was of 0.296 N/mm. This value of stiffness was not sufficient to transmit an average force greater than 17.5 N due to the limited stroke of the cable. In fact, the anchor point shifting ranged from 50 to 60 mm under a load of 17.5 N. Moreover, as qualitative data, the over-stretching of the glove was perceived as uncomfortable by the users, because of the localized forces exerted by the glove on the proximal part of the thumb. On the other side, the reinforced glove solution exhibited a stiffness value of 3.33 N/mm—about 10 times higher than the original glove: the average anchor point stretching under a load of 20 N was equal to 6.02 mm. The reinforced glove stiffness increase is the result of the combination of the sewn plastic 3D parts and of the Velcro straps that connect the two plastic parts. The smaller displacement of the glove translates into a more distributed load on the hand surfaces, thus resulting in a more comfortable glove.

### 3.2. Isometric Test

**Figure 8:**
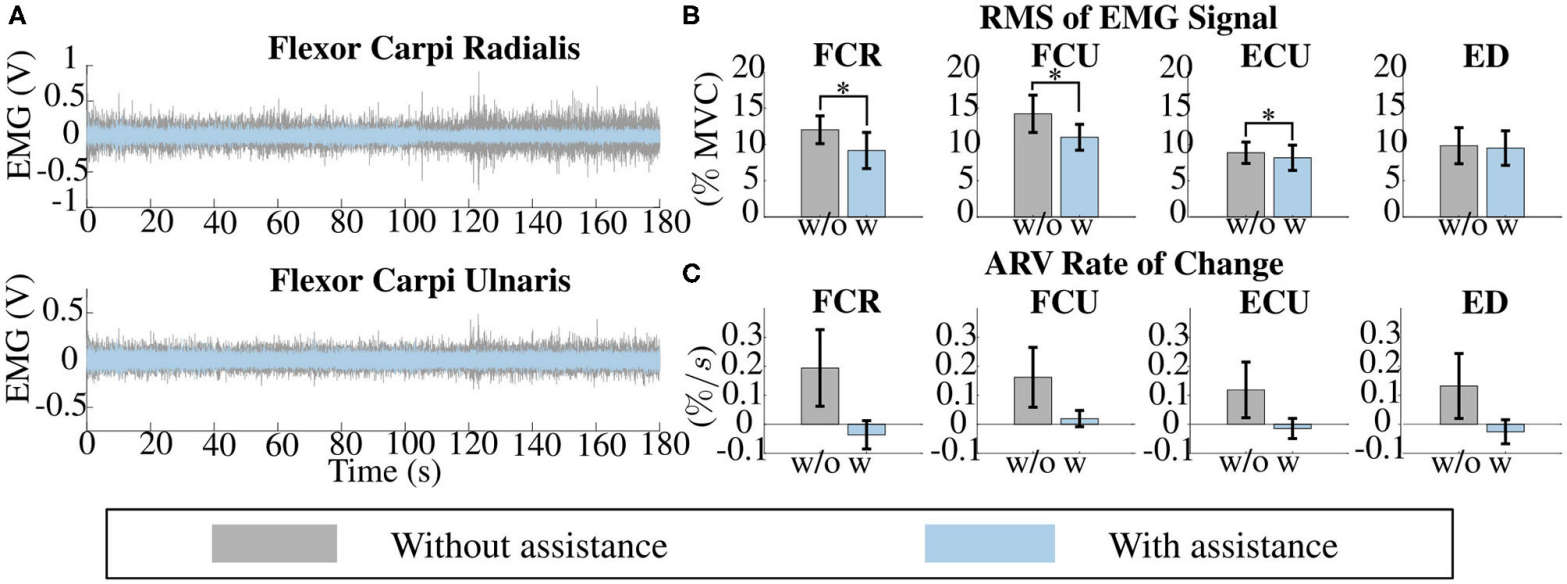
Fatigue analysis. **(A)** The raw signal of the electromyography (EMG) of the flexor carpi radialis (FCR) and of the flexor carpi ulnaris (FCU) of one subject, during the isometric task, for both the unpowered (gray) and powered condition (light-blue). **(B)** The overall mean of the 4 muscle' EMG signals, averaged over subjects, indicate that the assistance from the exosuit reduces the subject's muscular activity when a static holding of a load is performed. FCR, FCU, and extensor carpi ulnaris (ECU) EMG reduction were statistically significant (*p* < 0.01). **(C)** The slope of the ARV of the EMG signals, averaged over subjects and expressed in percentage of its initial value. A steeper positive slope for the ARV indicate a faster onset of fatigue (Merletti and Parker, 2004). These indices confirm that wearing the exosuit reduces the onset of fatigue. Error bars show the standard error of the mean. *means data are statistically significant with *p* < 0.01.

The results of the isometric test highlight that the use of active exosuits reduces the muscular activity required by the user to hold a weight and that exosuits can delay the onset of fatigue. The overall mean of the four muscles' EMG signals, averaged over participants, are depicted in Figure 8B. Specifically, the muscular activity reduction due to exosuit assistance was statistically significant for the flexor carpi radialis (from 12 ± 0.38 to 9.11 ± 0.47% of MVC, *p* = 3.65 × 10^−4^), the flexor carpi ulnaris (from 14.2 ± 0.49 to 10.94 ± 0.36% of MVC, *p* = 5.64 × 10^−4^), and the extensor carpi ulnaris (from 8.84 ± 0.29 to 8.12 ± 0.33% of MVC, *p* = 0.0074). Instead, the slight reduction of the extensor digitorum EMG signal resulted not statistically significant (from 9.78 ± 0.48 to 9.44 ± 0.46% of MVC, *p* = 0.5610).

The onset of fatigue was evaluated by analyzing the slope of the ARV of the EMG signals, averaged over participants, and expressed in percentage of its initial value. The computed ARV indexes are depicted in Figure 8C, where a steeper positive slope for the ARV indicates a faster onset of fatigue as reported in Merletti and Parker (2004).

ARV values obtained without exosuit assistance are all positive, i.e., a positive slope, thus the onset of fatigue was spotted (FCR: 0.19 ± 0.13%/*s*; FCU: 0.16 ± 0.1%/*s*; ECU: 0.12 ± 0.096%/*s*; ED: 0.13 ± 0.11%/*s*). Conversely, the assistance of the wrist exosuit inverted the slope of the ARV of the flexor carpi radiali, extensor carpi ulnaris, and extensor digitorum, whereas it only lessens the positive slope of the flexor carpi ulnaris (FCR: −0.036 ± 0.049%/*s*; FCU: 0.02 ± 0.028%/*s*; ECU: −0.015 ± 0.035%/*s*; ED: −0.026 ± 0.041%/*s*). Despite these results being encouraging, they were not statistically significant according to the paired *t*-test (FCR: *p* = 0.15; FCU: *p* = 0.23; ECU: *p* = 0.24; ED: *p* = 0.23).

### 3.3. Dynamic Test

**Figure 9:**
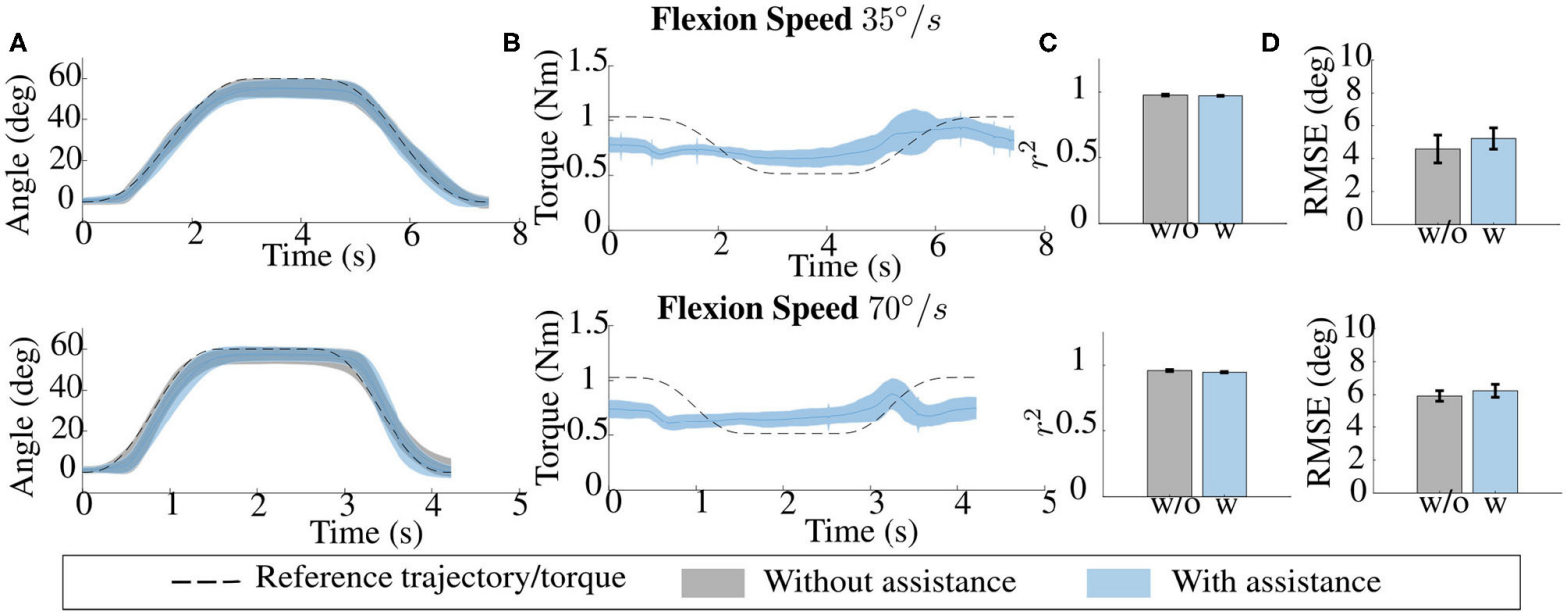
**(A)** Trajectories for the unpowered and powered conditions, averaged over repetitions, for one subject and at the two tested velocities (from top to bottom: 35 and 70 deg/s); as the velocity increases, the accuracy of the powered condition decreases. **(B)** The reference gravity torque and the assistance torque averaged over repetitions. **(C)** Average accuracy, measured through the coefficient of determination, *r*^2^, between the reference and measured trajectory of the wrist in the unpowered and powered condition. The overall mean, averaged over subjects, indicate that the assistance from the exosuit reduces a subject's capacity to follow a reference motion. Still, the absolute value of accuracy remain relatively high *r*^2^ = 0.971 ± 0.003 for 35°/*s* and *r*^2^ = 0.947 ± 0.005 for 70°/*s*. The same effect can be seen by analyzing in **(D)** the root mean square (RMS) error between the reference and measured trajectory of the wrist in the two conditions. In this case, the root mean square error (RMSE) slightly increased in the powered condition (with no significant statistical difference).

Figure 9 shows the effects of exosuit assistance on the users' capability of tracking the reference movement in the dynamic task. We evaluated the coefficient of determination, *r*^2^, between the reference and measured trajectory of the wrist in the unpowered and powered condition as a measurement of the average tracking accuracy (see Figure 9B). The overall mean averaged over participants indicates that the assistance from the exosuit reduces (without statistical significance *p* = 0.329) the participant's capability of following a reference motion. *r*^2^ indexes changed from *r*^2^ = 0.977 ± 0.006 for 35°/*s* and *r*^2^ = 0.96 ± 0.007 for 70°/*s* in the case of unpowered exosuit to *r*^2^ = 0.971 ± 0.003 for 35°/*s* and *r*^2^ = 0.947 ± 0.005 for 70°/*s* in the case of powered exosuit.

Despite the slight accuracy reduction, the absolute value of accuracy remains relatively high for both the tested speeds. The same effect can be seen by analyzing the RMS error between the reference and measured trajectory of the wrist in the two conditions (see Figure 9C). In this case, the root mean square error (RMSE) slightly increased in the powered condition (with no significant statistical difference *p* = 0.442). In particular, RMS error changed from *RMSE* = 4.57 ± 0.84° for 35°/*s* and *RMSE* = 5.92 ± 0.32° for 70°/*s* in the case of unpowered exosuit to *RMSE* = 5.21 ± 0.65° for 35°/*s* and *RMSE* = 6.24 ± 0.39° for 70°/*s* in the case of powered exosuit.

**Figure 10:**
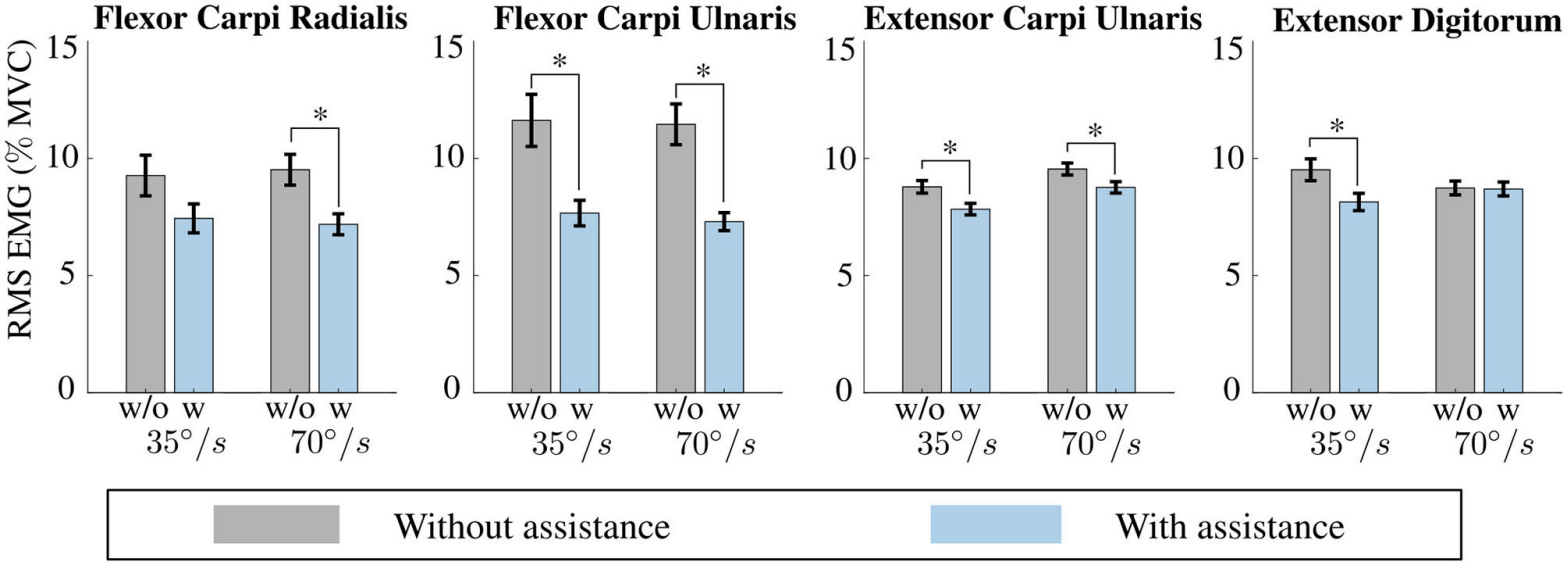
Reduction in muscular activity. The assistance provided by the exosuit reduced the muscular activity while performing a load lifting task at two different flexion speeds, 35 and 70 °/s. Reductions were statistically significant for all the muscles except for the flexor carpi radialis (FCR) at 35 °/s and for the extensor digitorum (ED) at 70 °/s. *means data are statistically significant with *p* < 0.01.

Figure 10 reports the effects of the exosuit assistance on the muscular activity while performing a load lifting task at two different flexion speeds. The main finding is that the soft exosuit reduces the EMG signals and is able to deliver assistive forces in accordance with the intended motion of the user, thus reducing the required effort. This experimental result was statistically significant for all the muscles except for the FCR at 35°/*s* and for the ED at 70°/*s*. Specifically, for the speed of 35°/*s* the RMS values of the EMG of the FCR reduced from 9.27 ± 0.87 to 7.44 ± 0.61% of MVC (*p* = 0.115); RMS of the FCU reduced from 11.62 ± 1.11 to 7.66 ± 0.55% of MVC (*p* = 0.0133); RMS of the ECU reduced from 8.78 ± 0.27 to 7.83 ± 0.25% of MVC (*p* = 0.114); and RMS of the ED reduced from 9.51 ± 0.47 to 8.14 ± 0.37% of MVC (*p* = 0.024). The significant reduction due to exosuit assistance was also kept in the high-speed case except for extensor digitorum muscle.

## 4. Discussion

In a working environment where collaboration between humans and robot is becoming increasingly common, exoskeletons represent the highest symbiotic solution. Safety and comfort are relevant aspects for a robotic device that needs to be worn. For this reason, exosuits are a promising paradigm to assist people in tasks that involve repetitive lifting, holding, or moving relatively small weight.

In this work, we implemented a wrist exosuit whose main requirements are lightness, ergonomics, modularity, and effectiveness when working in parallel with human muscles. To overcome the low force-transfer efficiency and uncomfortable pressure distribution of purely textile-based exosuits, we investigated the combination of soft fabric-based components with flexible 3D-printed plastic parts, sewn on the fabric. A key factor that drove our research was the evidence that a prominent contribution to the perceived discomfort comes from the localized pressure applied by non-ergonomic rigid supports on the human body. Moreover, the effectiveness of the exosuit assistance depends on its capability of efficiently transmitting forces/torques to the body. These considerations led us to the development of a lightweight glove with a reinforced path consisting of two 3D-printed hand-like shaped shell supports. These supports were designed as stiff as needed for the desired application; FEM analysis has been performed to define the shell thickness and stiffness. The design target was allowing a certain displacement for the exoskeleton to compensate for joint misalignment, with a relatively low static load transferred between the human and the exosuit. The desired stiffness should be low to avoid excessive discomfort but, at the same time, it should be sufficient to guarantee the functional requirement of bearing a cable tension of at least 50 N without compromising the motion and the force transmission principle. In particular, the transmission stiffness test was conducted to evaluate the difference between a complete soft solution and the flexible solution we proposed. From the test results, we acknowledged that a purely fabric-based soft orthosis cannot manage the tension values required to lift even relatively low weights (3 kg), as the upper limit is about 17.5 N, which already causes a displacement of the orthosis that impedes the exoskeleton and its actuation principle from working correctly. On the contrary, the new hybrid solution proposed, made up of a combination of soft orthosis and sewn 3D-printed plastic reinforcements, can withstand up to 50 N of cable tension while effectively transferring torque to the joint, leading to a 10-fold increase in stiffness of the transmission. Furthermore, participants did not report discomfort during the experiments, when wearing the reinforced glove, in spite of higher loads.

As a secondary outcome, in this study we evaluated the effects of the proposed exosuit on the activation and onset of fatigue of the major muscles involved in flexing and extending the wrist, using an indirect force controller for gravity compensation. Statically holding a load with the assistance from the powered exosuit lowered the muscular effort by an average 26.16% and 18.11% for the flexor carpi radialis and the flexor carpi ulnaris, respectively, compared to wearing the device but not receiving assistance. A slight but not substantial change was observed in extensor muscles.

The impact of the exosuit on the reduction of muscular activity is comparable to the one obtained with other soft wearable devices (e.g., O'Neill et al., 2017; Chiaradia et al., 2018a). The relative reductions in muscular activation are lower than in other studies, probably because of the effect of cross-talk in the EMG measurements, between wrist and finger flexor muscles; the latter were not supported by the exosuits but were still required to hold the additional mass used in the protocol. This warrants for further investigation, using measurement techniques with higher spatial resolution (e.g., needle electrodes), signal processing methods to isolate sources of EMG activity, or designing a protocol that does not involve grasping. Still, our results are significant for 3 muscles out of 4: while the reduction for the two flexor muscles can be considered an expected result, and this is not the case for the reduction of the ECU. We speculate that this would be due to the need for stabilizing the added mass by stiffening the wrist through co-contraction. The tensioned exosuit might increase stability, acting like a splint and thus reducing the need for co-activating antagonist muscles. Further investigation is needed to confirm this theory. A direct corollary of the reduction in muscular activity is the delay in the onset of muscular activity, measured by the increase in magnitude of the EMG amplitude over the course of a loaded isometric task. This confirms that the device is working in parallel with the wrist flexors and is encouraging evidence for the functional effects that this device could have in industrial settings—allowing for a higher number of loading cycles—or clinical scenarios—enabling patients to perform physical training sessions at a higher intensity or volume.

A similar effect was observed during dynamic movements, where the exosuit followed its wearer's movements while compensating for gravitational forces. The activity of the FCR and FCU was reduced when wearing the device, compared to an unpowered conditions, in both of the tested velocities. Average relative reductions were −16.43 and −26.85 % for the FCR and FCU, respectively. This was achieved with a small effect on kinematics. Compared with our previous work on the elbow (Xiloyannis et al., 2019a), the deterioration for the wrist exosuit is smaller—from 0.91 to 0.80 in the elbow exosuit, whereas from 0.96 to 0.95 in the proposed wrist exosuit. One of the causes that mostly affect movement accuracy is the transparency of the exosuit. Specifically, the transparency we are referring to is not a purely mechanical one, but reflects the capability of the controller to follow the human movement with little unwanted interaction forces. Therefore, the force-tracking performance of the admittance controller plays an important role alongside the hardware characteristics of the device. With this in mind, we can assert that the unmodeled and configuration-dependent friction in the Bowden cable transmission is mainly responsible for the degradation in motion accuracy, although this effects was mitigated by the use of a positive feedback term in the control and by the high-gain low-level velocity loop.

It is worth highlighting that the muscular activity index results of this work were obtained with a procedure fundamentally different from the one used in other comparable studies (e.g., Simpson et al., 2017; Thalman et al., 2018): the admittance controller that we designed allowed the suit to continuously move in concert with its wearer while delivering an assistive torque. The other studies listed here lacked an intention–detection strategy. The robot was triggered to apply a predefined torque or trajectory, regardless of the intention of the wearer: during the evaluation, the participant was simply asked to relax. The motion intention strategy used in this work is basically the same one used for our previously developed elbow exosuit (Xiloyannis et al., 2019a), but in this work the feed-forward velocity contribution was generated by using directly gyroscope angular speed measurements rather than angular speed estimates from encoder reads. We tried to overcome the issue related to the significant effort at initiation of movement experienced in the elbow suit. Clearly, a solution that does not use information that is directly related to the user motion intention (e.g., EMG signals) but that is based on motion's effects (e.g., the flexion speed of the wrist) suffers from time delay, and thus it is not able to completely overcome the mentioned problem. Nevertheless, the IMU was positioned on the most distal part of the suit to take advantage of the high compliance of the system. What is more, wrist flexion speed was not obtained from position differentiation, thus discretization related effects were avoided. Thus, the controlled system is made backdrivable by the proposed indirect force control, namely an admittance control with a feed-forward speed-proportional contribution.

We acknowledge that, despite the encouraging results of the technology and analysis presented here, there are a number of limitations to this work. First, the small sample size of our analysis does not allow for generalizable results on the effect of the wrist exosuit on the biomechanics of human movement. The results on the amplitude of surface EMG activity of the muscles on the forearm, moreover, despite careful positioning of the electrodes, warrant caution in their interpretation because of possibility of cross-talk from finger flexors/extensors. The baseline condition used in this study was an “exosuit on but unpowered” one, chosen for practical reasons. A “no exosuit” condition would allow a more objective assessment of the effect of the device in real-world scenarios. Moreover, the device has still some limitations; the Bowden cable transmission offers the possibility of remotely locating the actuation, decreasing mass and inertia of moving parts and resulting in a more transparent system. However, a considerable friction is involved with the use of this transmission system; this friction, if not properly managed by a fast and responsive controller, causes a variable disturbance transferred from the exoskeleton to the human during motion. Even if of little entity, this disturbance partially compromises the fluidity of motion for the wearer. However, accurately estimating the friction due to Bowden cable can be complicated, as it depends on a multitude of factors: sheath friction coefficient, sheath's wrap angle, and cable tension. Therefore, further improvements on the controller are needed to manage this feature of the transmission. There is room for improving the effects of the device on human movements through a more accurate model of the cable geometry and mass of the hand. The model used here was based on anthropometric data and could be augmented through identification techniques (Just et al., 2020). The presented device assists only the wrist flexion and this can limit the amount of assistance for some lifting tasks that would benefit from assistance torque at the ulnar/radial deviation degree of freedom. The presented prototype device aims to validate the viability of the new design approach and of the actuation solution.

Our work attempted to further improve transmission efficiency and comfort at the physical HRI through the design of a hybrid, personalized approach. This method could be taken a step further by not only mapping the morphological but also the biomechanical properties of human tissues: Sengeh and colleagues, for example, designed a prosthetic socket for trans-tibial amputees with variable 3D stiffness, designed to mirror the stiffness of the residual limb and avoid peaks of pressure (Sengeh and Herr, 2013; Petron et al., 2016). A spatially varying stiffness profile was similarly used to improve ergonomics of the dorsal attachment of a hand exoskeleton (Varghese et al., 2018). Finally, active cuffs Choi et al. (2019) or designs that exploit a smart topology of the fabric fibers to uniformly distribute forces on the underlying soft tissues Witherspoon and Kernbaum (2018) are potentially auspicious paths.

Concerning mechanical properties and requirements, this exosuit allows the wearer to lift small weights up to 3 kg, which does not cover the range of weight typical for certain industrial and logistics application, where a worker needs to lift up to 20 kg boxes. Nevertheless, soft “hybrid” exosuits that implements the concept of “stiff as needed” and based on fabric materials with ergonomic reinforcements made up of 3D-printed plastic—or even small metallic parts—could be the compromise that allows these assistive devices to make an important step forward toward a more significant adoption of exoskeletons in real-life scenarios.

## Data Availability Statement

The raw data supporting the conclusions of this article will be made available by the authors, without undue reservation.

## Ethics Statement

Ethical review and approval was not required for the study on human participants in accordance with the local legislation and institutional requirements. The patients/participants provided their written informed consent to participate in this study.

## Author Contributions

DC, LT, and MX designed the exosuit and developed the controller. DC and LT performed the experiment. All authors designed the experiment, analyzed and interpreted the data, prepared the manuscript, provided critical feedback on the manuscript, and read and approved the final manuscript.

## Conflict of Interest

The authors declare that the research was conducted in the absence of any commercial or financial relationships that could be construed as a potential conflict of interest.
