# Supplementary material for: An Assistive Soft Wrist Exosuit for Flexion Movements With an Ergonomic Reinforced Glove
Source: Front Robot AI. 2021 Jan 18;7:595862. doi: 10.3389/frobt.2020.595862 (PMC7848217; doi:10.3389/frobt.2020.595862)
Supplement: Supplementary file 1 [file Data_Sheet_2.PDF]

Supplementary Material

1 SUPPLEMENTARY DATA

2 SUPPLEMENTARY TABLES AND FIGURES

2.1 Figures

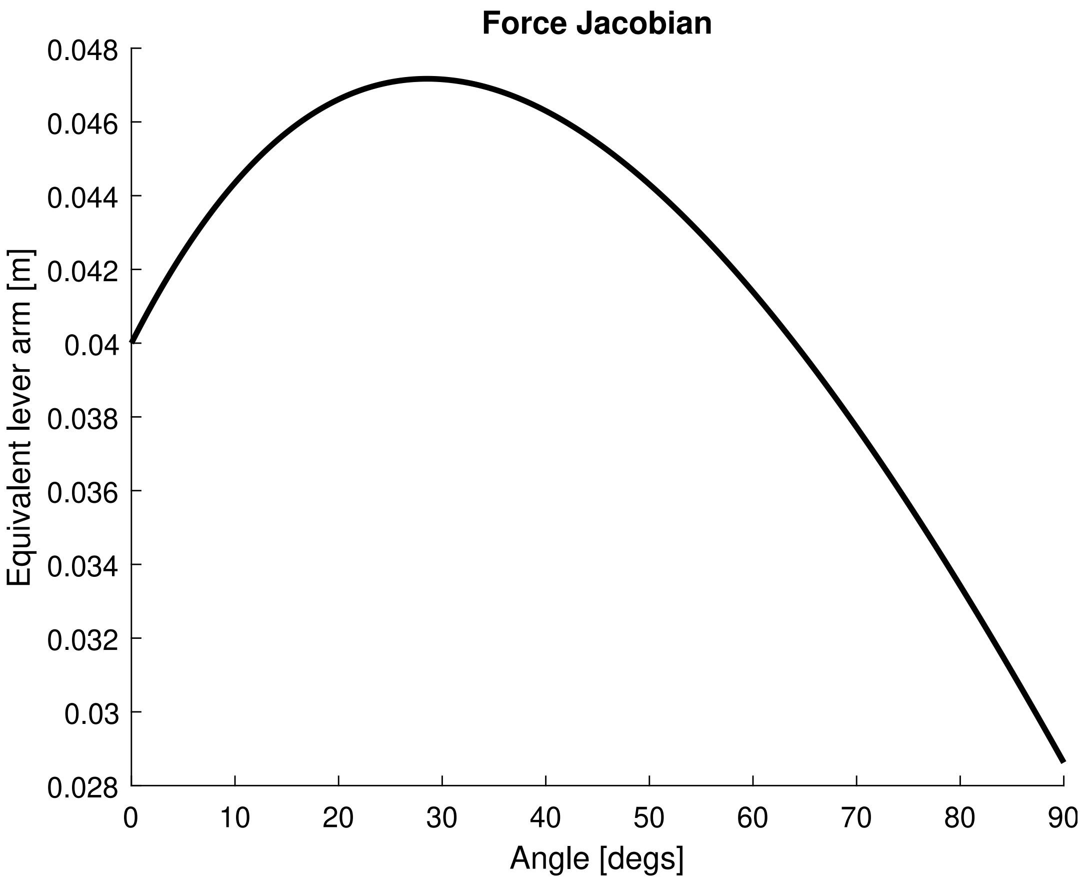

Figure S1. Force Jacobian: the plot represents the value of the equivalent lever arm of the tendon in the wrist flexion ROM, depending on the flexion angle because of the device's geometry. Importantly, there is no singularity in the wrist flexion ROM.
